# Upper Extremity Reconstructions

**DOI:** 10.1080/135771402753667691

**Published:** 2002-05


### UPPER EXTREMITY RECONSTRUCTIONS

### ORAL PRESENTATIONS

### F54 Resection in Primary Malignant Scapula Tumours

A.H.M. Taminiau, R. Bloem, S. Dijkstra, P.C.W. Hogendoorn
*Leiden University Medical Center, Leiden, The Netherlands*

Limb salvage procedures in primary bone tumours of the scapula offer attractive prospects regarding function and aesthetics. What procedure depends mainly on the site/extension, stage/diagnosis. In scapular resections the glenoid plays the key role. This retrospective study shows single centre Results regarding outcome.
*Methods:* From October 1983–February 1998 thirty-three patients presenting with primary malignant bone and soft tissue tumours of the scapula were treated in LUMC. Tumour type: chondrosarcoma (17), Ewing (7), MFH (5), osteosarcoma, leiomyosarcoma, rhabdomyosarcoma, synoviosarcoma either one. Patient gender (male 17, female 16). The mean age 35.4 (10–69 y). Mean follow-up 9 years (4–18 years).
*Results:* Four patients had only chemotherapy and radiation therapy (Ewing 3, MFH 1). In 30 patients the procedures performed were resection (28) and fore quarter (2). The scapula resection consisted in corpus (16), total scapula (6), acromion (3) and glenoid (3). Reconstruction was performed by allograft [total scapula (2), radius/glenoid (2)] or prosthesis (total scapula (3), glenoid (1) and Tikhoff-Linberg (1). Oncological outcome: recurrence 5 [chondrosarcoma (4), rabdomyosarcoma (1)]. Treatment recurrence resection (3), forequarter (1). Disease free survivors 22; tumour related death 6 [4 Stage IIIB, Ewing (2), MFH, rhabdomyosarcoma] alive with disease 4 [chondrosarcoma (4)], death unrelated to tumour 1 [chondrosarcoma]. Complications reconstruction: No complications in partial resections of the scapular body (soft tissue reconstruction). Both total scapula allografts had complications: resorption and fracture (1), undisplaced fracture (1). Radius/glenoid reconstruction: resorption (1). Prosthesis: no complications [except traumatic humeral fracture(1)]. No infections/luxation. All shoulders were stable including the allograft with resorption. 4 of 7 Ewing (all had chemotherapy + radiotherapy) survived. 3 out of 4 survivors of the Ewing were treated by resection (one total 2 partial). If the glenoid was preserved functional Results are overall good. All patients had stable shoulder functions. The more scapula (spina scapula) and muscle remained the better function. Acromion resection resulted in normal function in all. Glenoid reconstruction (allograft, prosthesis) resulted in stable but restricted shoulder, good elbow hand function in all. In all patients the cosmetic appearance was good or excellent.
*Conclusion:* Resection of part/total scapula might result in a stable often good shoulder function especially if the glenoid can be preserved.

### F55 Upper Limb Reconstruction in Bone Sarcomas of Childhood

M. Manfrini, S. Giacomini, L. Campanacci, D. Mellano, M. Mercuri
*Istituti Ortopedici Rizzoli, Bologna, Italy*

Oncological and functional Results were assessed in 75 children (<15 yrs) with upper extremity primary sarcomas treated by limb salvage from 1980–2000. Diagnosis was osteosarcoma in 55 patients (44 stage IIB, 11 stage III), Ewing's sarcoma in 18 (16 localized, 2 metastatic cases), and malignant fibrous histiocytoma in 2. All patients received chemotherapy. Megaprostheses were implanted in 37 cases: 33 proximal humerus, 3 total humerus, 1 distal humerus. 6 patients had osteoarticular allografts: 2 proximal, 2 total and 1 distal humerus, 1 distal radius. A methylmetacrylate spacer and a Kuntscher nail were inserted as proximal humerus spacer in 9 patients. For reconstructing the proximal humerus (11) or the distal radius (3) with a potential growth, a vascularized proximal fibula autograft (VPFA) was used in 14 young children (range 2–7 yrs). Various intercalary grafts (allografts, simple or vascularized autografts) were used in 9 patients (5 humerus, 3 radius, 1 ulna). At a mean follow-up of 92 months, 46 patients (61%) are disease-free. The oncologic outcome showed a striking difference in prognosis of localized Ewing' sarcoma (75% CDF) compared to stage II osteosarcoma (45% CDF). Local recurrence rate was 8% (6/75); but fell to 3% (2/62) with wide margins and increased to 31% (4/13) after inadequate margins. According to MSTS evaluation system, both endoprosthesis and spacer reached acceptable Results in shoulder reconstruction but the application of VPFA improved the function, allowing the biological growth of the arm. Great changes in reconstructive techniques characterize the evolution of this series. In the period 1980–1990, the most common device was a synthetic implant (88%). Recently, the expanded indication to biological reconstructions in prepubertal age reduced the indication for artificial implants, improving in younger patients the growth and function of the reconstructed upper limb.

### F56 Upper Extremity Reconstruction: Is Limb-salvage for Osteosarcoma of the Proximal Humerus a 'SAFE' Procedure?

S. Bielack[1], S. Flege[1], R. Kotz[2], V. Ewerbeck[3], G.U. Exner[3], U. Heise[4], W. Winkelmann[3], H. Jurgens[1]
[1]*University Children's Hospital, Muenster, Germany,* [2]*Vienna University Hospital, Vienna, Austria,* [3]*University Hospital, Heidelberg, Germany,* [4]*Orthopedic Private Practice, Hamburg, Germany*

*Objectives:* The proximal humerus is the third most frequent site involved by osteosarcoma. The primary aim of this analysis was to determine whether affected patients could safely retain their affected limb.
*Methods:* All patients registered into a neoadjuvant COSS-study between the end of 1979 and June 1998 with the diagnosis of a previously untreated (except primary surgery) high-grade osteosarcoma of the proximal humerus who had achieved a macroscopically complete surgical remission at the primary site during 1st line combination therapy were evaluated for type of surgery used, local control and survival.
*Results:* 162 eligible patients were identified (median age: 14.5 years (range 2–66); 89 male, 73 female; 75 tumours <1/3 and 86 $^3$1/3 of the humerus (1?); localized 142, primary metastastatic 20). Definitive surgery was ablative in only 29 patients (18%, incl. 2 secondary amputations), while the limb was salvaged in 133 (82%). With a median follow up of 4.2 years (.15–21.1), 91 patients survived, for a 5 year actuarial survival rate of 58%. Altogether, 11 patients (6.8%) experienced a local recurrence (median time from surgery: .8 years, range: .4–2.8). All local failures occurred after limb-salvage surgery (11/133, 8.3%), usually in the soft tissues. Intralesional or marginal margins were reported for 9 tumours, of which 3 relapsed locally. There were only 2 local relapses among 82 tumours known to have responded well (<10% viable tumour) to preoperative chemotherapy (2.5%), but 8 among 63 poor responders (12.7%). The local failure rate after limb salvage in poor responders was 17.4% (8/46). Only one of the 11 patients with a local recurrence is still alive.
*Conclusion:* Amputation is rarely used for proximal humerus osteosarcomas. Limb salvage seems to be a safe alternative for most tumours which respond well to preoperative chemotherapy.

**F57 Cemented Modular Prosthesis in the Proximal Humerus: Long Term Follow-up**

M. Donati, G. Bianchi, P. Ruggieri, M. Mercuri
*Istituti Ortopedici Rizzoli, Bologna, Italy*

From February 1975 to December 1990, 144 patients have been operated for a musculo-skeletal tumour of the shoulder girdle with resection and reconstruction using a modular cemented prosthesis. The MRS prosthesis is assembled in three parts with a ball shaped rotating head stitched to the glenoid and acromium. Seventy patients died and 3 patients were lost to follow-up: 71 achieved a follow-up more than 10 years (123–259, av 175 mo). Age ranged from 9 to 73 years (mean 29.7). The resection was intrarticular in 44 cases, in 12 more the glenoid was resected along with the proximal humerus (extrarticular resection) and in 15 cases we performed a Tikhoff–Lindberg procedure. Infection occurred in 7 patients (10%) from 1 to 144 months (median 12 mo): in 6 patients prosthesis removal was needed to achieve healing. Mechanical complications were present in 19 patients (27%): 15 (21%) had prosthetic head instability (5 surgically treated), 2 breakage of the prosthetic stem and 2 prosthetic disassembly. A detailed roentgenographic analysis has been developed to better define the long term course of the cement bone interface. Only 3 aseptic stem loosening were detected at 1, 3 and 11 years (after a supercondylar fracture occurred 8 months before). Eighteen patients were reoperated (25%); in 4 cases with minor surgery. Failure of the system occurred in 11 cases (15%). The long durability of this cemented prosthesis has been demonstrated with very few cases of stem loosening in the early follow-up time. The problem of a good prosthetic head suture is still under concerning particularly in Tikhoff–Lindberg procedure.

### POSTER PRESENTATIONS

**F58 Shoulder Function Preservation in Proximal Humerus Osteosarcoma**

E.N. Noain, M. San-Julian, P.D.R. Dfaz de Rada
*Clinica Univeristaria de Navarra, Pamplona, Spain*

The objective of this paper is to present proximal humerus osteosarcoma case in which Ca adell's technique could be successfully used.

*Methods:* A 12 year old girl affected by an osteosarcoma of the proximal humerus without epiphyseal involvement, was operated on according to the Ca adell technique (epiphysiolysis before excision of the tumour) in order to preserve the shoulder joint. The proximal pins of the external fixation were placed in the anterior side of the epiphysis, due to the particular morphology of this growth plate. The distal pins were placed in the posterior part of the distal humerus, in order to avoid radial nerve damage.

*Results:* After 8 days of distraction, epiphysiolysis was done. The tumour was removed and the reconstruction was carried out with an intercalary allograft. Twelve months later the shoulder joint has an almost complete motion range.

*Discussion:* Proximal humerus is the third most frequent location of malignant bone tumours. There are several reconstruction techniques for these tumours: vascular fibula, osteoarticular allografts, prosthesis. ... All these techniques preserve the hand and elbow function, while shoulder function usually remains poor. In most cases the implant used acts as a simple spacer. Epiphysiolysis before excision allows to preserve the shoulder joint in selected cases. The rotator cuff could be also be preserved, and the functional Results are superior to other reconstruction techniques in this location. The placement of the pins of the external fixation should be carefully done. The growth plate remains together with the preserved epiphysis, although this is not too much important in this location.

**F59 Outcome of Endoprosthetic Replacement for Proximal Humeral Metastasis**

J. Borg, S. Mahroof, J. Pringle, P. Unwin, T.W.R. Briggs, S.R.C. Cannon
*Royal National Orthopaedic Hospital, London, United Kingdom*

Whilst the treatment of primary tumours of the proximal humerus with endoprosthetic replacement is generally accepted, that of metastatic lesions remains controversial. Poor prognosis has been accepted early and such patients are treated with radiotherapy and skeletal stabilisation where possible. Endoprosthetic replacement for proximal humeral metastasis was performed on 10 patients between 1993 and 2001. 4 patients presented with a pathological fracture with no known primary. In all the primary lesion was known only in 50% of patients. The most common underlying pathology was renal carcinoma (clear and adenocarcinoma) in 5 patients and one case each of bronchial, leiomyosarcoma and endometrial carcinoma. 1 case was adenocarcinoma from unidentified primary. Two patients had had previous skeletal stabilisation prior to the prosthesis. Two patients died of their disease and one patient died of post-op renal failure. The average follow up for the other patients is 26 months. The endoprosthesis used was cemented in the early cases and HA-coated in the more recent ones. There was one case of dislocation and no cases of implant failure of infection.

*Conclusion:* In our experience endoprosthetic replacement should be considered for proximal humeral metastasis. This form of treatment is preferred to skeletal stabilisation in well selected cases.

**F60 Autobiologic Reconstruction of the Shoulder with Preserved Joint Function. A Case Report with 2.5 Years Follow up.**

K. Berlin, P. Bergh, H. Mark, J. Lundberg
*University of Goteborg, Goteborg, Sweden*

A 6-year-old girl presented with pain in her left arm in July 1999 after a minor trauma. Radiograms showed an osteolytic destruction with a pathologic fracture in the metaphysis of the proximal humerus. A core biopsy revealed a stage IIB telangiectatic osteogenic sarcoma. Preoperative chemotherapy (ISG/SSG I-protocol) was commenced and consecutive MR-examinations revealed a good tumour response. The objective of this study is to evaluate the Results of a joint-preserving autologous reconstruction of the shoulder joint. Operative treatment. In November 1999 the tumour was resected including the proximal 10 cm of humerus. A microvascular fibula graft was dissected including the insertion of the lateral collateral ligament. The graft was inserted into the distal humerus. The fibula periosteum was draped over the osteotomy site. The origin of the long tendon of the biceps brachi muscle was sutured to the lateral collateral ligament of the fibula. The rotator cuff was preserved and sutured around the fibula head to regain a capsule-like enclosure of the fibula head against the glenoid. The fibula head was transfixed to the glenoid during 10 days and the patient had a thoracobrachial orthosis for 3 months. Chemotherapy was concluded 10 months postoperatively.

*Functional Results:* Scintigraphy and radiograms 4.5 months postoperatively showed healing at the osteotomy site. The entire graft was fully vascularized. At 18 months the patient was able to ski and could elevate her arm to 90 degrees. With a trick motion she could elevate her arm up to 160 degrees. At 30 months she is able to lift 2 kg from her waist to 160 degrees elevation. The MSTS-score is 27 (90%).
*Conclusion:* The functional and oncologic Results so far are promising. The reconstruction of the shoulder joint as described is crucial for the good functional outcome of the operation.

**F61 Postresectional Arthrodesis and Endoprosthesis of the Shoulder Preliminary Results**

B. Sbutega, N. Lujic, R. Radivojevic
*'Banjica' Institute for Orthopedic & Surgery, Beograd, Yugoslavia*

Authors pointed out two different approaches in the treatment of proximal humeral bone tumours. After surgical-staging-procedures 11 patients underwent wide resection followed with two modalities of reconstruction. In five cases reconstruction was performed by arthrodesis. In all, diagnosis was proved after biopsy as; malignant-chondroblastoma, telangiectatic osteosarcoma, synoviosarcoma, chondroblastoma and Ewing-sa. The length of resections were; 8 cm, 15-cm, 12-cm, 9-cm and 22-cm. All patients are with NED. As far as complications are concerned in one case it was stem-loosening and in other prolonged-healing. Sex distribution; male 1/female 4. Age 12–22 years. Chemotherapy in two cases and follow up four years mean time 2.5 years. Functional evaluation six months post op as following; 70% – good, 70% – good, 56.6% – satisfied, 76.6% – good and 56.6% – two years later 63.3% – satisfied. On the other hand in 6 patients after resection reconstruction was performed by prosthesis. According to biopsy diagnosis was proved in two cases as Ewing sa, osteosarcoma, ABC, Chondrosarcoma and GCT II. The length of resection were, 11 cm, 14 cm, 10 cm, 12 cm, 14 cm and 13 cm related to margin in four cases wide and in two marginal. Average follow up is 6 years; two are NED, in one case mets and death after a year post op and in others local recurrences. Sex ration was 3/3. Age 19–21, average 28 years. According to complications in all instances surgery was performed 2,16 times. Chemotreatment were applied in three cases and fatal outcome was in three patients. Functional evaluation was as follows: 36% – bad, 50% – satisfactory, 33% – bad, 66% – good, 60% – satisfactory and 30% – bad. According to onco – functional Results resection arthrodesis is absolute the real option and the only one advantage of prosthesisis better x-ray picture.

**F62 Limb-salvage Operations in the Treatment of Shoulder Girdle Tumours**

M.D. Aliev, V.A. Sokolovskyi, A.A. Khamdamov, O.G. Zimina
*Blokhin Cancer Center, Moscow, Russian Federation*

*Objective:* To analyze the treatment Results in 75 patients with tumours of the shoulder girdle, treated between 1992 and 2000.
*Methods:* There were 38 males and 37 females. The mean age was 45 years (14–30). 68 patients (91%) had tumours located in the humerus and 7 (9%) in the scapula. Histology was osteosarcoma 8, chondrosarcoma – 27, parosteal sarcoma – 6, giant cell tumour-20, MFH – 4, and benign tumour – 10. The following operations were performed: the resection of proximal humerus with endoprosthetic replacement – 22 (29,3%), Tikhoff-Lindberg procedure – 21 (28%), segmental resections with autoplastics – 9 (12%), segmental resections with alloplastics-11 (14,7%), segmental resections without reconstruction – 6 (8%), marginal resection – 3 (4%) and curettage – 3 (4%).
*Results:* Local recurrences occured in 17 pts.: after endoprosthetic replacement-27%, after segmental resections with auto-and alloplastics-70%, after Tikhoff-Lindberg procedure-19%. Postoperative complications (fracture and graft resorbtion) occured in 8 (40%) of 20 pts. treated by segmental resections with auto- and alloplastics.
*Conclusion:* Limb salvage operations are indicated in the treatment of shoulder girdle tumours.

**F63 Upper Extremity Reconstructions in Malignant Humeral Bone Tumours in Children**

W.W. Wozniak, I.K. Kiendrak, E.R. Rogowska,
M.R. Rychlowska, M.K. Kuczabski
*Nat. Research Institute of Mother&Child, Warsaw, Poland*

Between 1985 and 2000, about 350 children with malignant bone tumours were treated at the National Research Institute of Mother and Child in Poland. In 22 patients (13 girls, 9 boys) aged 5–18 years (4 under 10 years) with upper limb tumour – Osteosarcoma (15), Ewing's Sarcoma (4), Chondrosarcoma (2) and Fibromatosis aggresiva (1) limb-salvage operation were performed. Most common localization-proximal humerus (16). After neoadjuvant chemotherapy it was possible to perform excision of tumour with reconstructive limb surgery in all patient. Wide resection of the tumour (a 7,5- to 23-cm-long fragment of humerus) was performed, entire humerus was removed in 1 case. According to diameter and localization of tumour, technical possibilities and age of patient, different Methods of reconstruction have been performed: 'clavicula pro humero' (according to Winkelman) (5); allograft reconstruction (9) – 5 with Kuntsher nails and 4 – with Howmedica endoprothesis; autograft reconstruction-fibula (1); endoprothesis (5) – 4 Mutars endoprothesis, 1 total humerus replacement endoprothesis Howmedica; Zimmer's elbow endoprothesis (1); without reconstruction (1). 12 patients had poor Results because of bone graft fracture (8), local recurrence (3), internal stabilization complication (1) and re-operation had been necessary as local revision and internal stabilization (6), limb amputation (3), and Mutars endoprothesis implantation (3). In all patients the overall functional Results achieved as excellent in palm and forearm function (temporary radial nerve palsy (3)), the movements in shoulder joint (12) and elbow joint (3) have been limited. 19 patient of the 22 have been surviving for 16 years to 16 months (8 patients – 5 years after the end-of-treatment), 3 patients died. Functional Results were satisfactory but there were some complication related to the reconstruction, although limb salvage surgery has surpassed amputation as the treatment for malignant humeral bone tumours in children.

**F64 Cancellous Bone Graft Reconstruction for Tumours of the Hand**

S. Mahroof[1], D. Evans[2], J. Pringle[1], S.R. Cannon[1], T.W. Briggs[1]
[1]*Royal National Orthopaedic Hospital, London, United Kingdom,*
[2]*Windsor Hand Clinic, Windsor, United Kingdom*

*Objective:* Although preservation of function following excision of phalangeal or metacarpal bone tumours has been achieved using autologous or allogeneic cortical bone grafts, complications are not uncommon. An alternative technique is described in which the use of autologous cancellous bone from the iliac crest to provide both structural support and a stimulus for bone formation, with potentially less morbidity.
*Methods:* Three patients with low grade chondrosarcomas (distal phalanx, proximal phalanx and first metacarpal) and one patient with a giant cell tumour (fourth metacarpal) underwent primary tumour excision followed by reconstruction using a block of cancellous bone harvested from the iliac crest. Graft size was determined preoperatively using plain radiography, and grafts were stabilised intraoperatively using either K-wires or Swanson arthroplasty. Functional outcome was evaluated using the Sollerman hand function test at six months postoperatively.
*Results:* Two patients who underwent metacarpal reconstruction demonstrated consolidation of the graft at eight and six weeks postoperatively, and had excellent hand function at six months (Sollerman scores 79/80 and 79/80). Although the two phalangeal tumours were thought to be enchondromas preoperatively, histological examination confirmed them both to be chondrosarcomas, and these two patients therefore underwent subsequent amputation to achieve tumour clearance. In spite of this, both grafts demonstrated radiological union. All patients reported minimal pain from donor sites postoperatively.
*Conclusion:* Following excision of bone tumours in the hand, reconstruction can be effectively achieved using autologous cancellous bone grafts as an alternative to cortical bone, with potentially faster rates of union and resistance to resorption, while maintaining structural support. This technique may avoid the complications of both autografts (donor site pain and immobility) and allografts (rejection and non-union).

**F65 Limb-sparing Resections of the Shoulder Girdle. A Long-term Follow-up Study**

J. Bickels[1], J.C. Wittig[2], Y. Kollender[1], R. Oren[1], K. Kellar-Graney[2], M.M. Malawer[2], I. Meller[1]
[1]*Tel Aviv Sourasky Medical Center, Tel Aviv, Israel*
[2]*Washington Cancer Institute, Washington, USA*

*Introduction:* Limb-sparing-surgeries around the shoulder-girdle pose a surgical difficulty because tumours arising in this location are frequently large at presentation, juxtaposed to the neurovascular-bundle, require enbloc resection of proportionally significant amounts of bone and soft-tissues, and necessitate complex-resection and reconstruction. Between 1980 and 1997, the authors performed 134 limb-sparing-resections around the shoulder-girdle; all patients were followed for a minimum of 2 years. On the basis of their experience, the authors outline the guidelines for surgical-management of a large-tumour of the shoulder-girdle.
*Methods:* There were 71 male and 63 female patients who ranged in age from 9 to 90 years who were diagnosed as having 110 primary malignant, 12 metastatic, and 12 benign-aggressive-tumours. Preoperative-evaluation emphasized physical-assessment of the anatomic-relation of the lesion to the major neurovascular-bundle of the upper-extremity, and availability of uninvolved muscles for soft tissue reconstruction. This was followed by comprehensive imaging studies. Surgery entailed the use of the utilitarian shoulder girdle incision for tumour exposure. Endoprostheses were used for reconstruction and included 92 proximal humerus and nine scapular implants. All patients were followed up for a minimum of 2 years. Functional evaluation was done according to the American Musculoskeletal Tumour Society System.
*Results:* Function was estimated to be good or excellent in 101 patients (75.4%), moderate in 23 patients (17.1%), and poor in 10 patients (7.5%). Complications included 13 transient nerve palsies, two deep wound infections, and one prosthetic loosening. Local tumour recurrence occurred in five of 103 (4.9%) patients with primary sarcomas of the shoulder girdle.
*Conclusion:* Detailed preoperative evaluation and surgical planning are essential for performing a limb-sparing resection around the shoulder girdle. Local tumour control, associated with good functional outcome and prosthetic survivorship is achieved in the majority of the patients.

**F66 Claviculectomy for Bone Tumours**

Y. Kollender, N. Mayilvahanan, M.M. Malawer, J. Bickels, O. Merimsky, J. Issakov, G. Flusser, A. Nirkin, N. Marouani, I. Meller
[1]*Nat. Unit of Orhtopedic Oncology, Tel Aviv Sourasky Medical Center, Tel Aviv, Israel*

The clavicle is the only bone which connects the upper limb to the axial skeleton. No significant defect in the shoulder function has been described following clavicular resections. It is considered as an accessory baggage of the skeleton. Total or partial excision of the clavicle has been advocated for many neoplastic and non-neoplastic conditions. Between 1991 and 1998 twelve patients underwent claviculectomy for various tumours. Histopathologically Ewing's sarcoma was the commonest. Age ranged from 4 to 70 years (mean 30 years). 8 patients were males and 4 females. Follow up time was between 3 to 10 years (mean 5 years). A new classification system for claviculectomy has been evolved based on the extent of the resection. Functional Results were analysed using the AMSTS scoring system. Functional outcome was excellent in 5 cases and good in 7 cases. There was no evidence of disease in any of the patients during follow up period.
*Conclusion:* Partial or total claviculectomy can be successfully employed for bone tumours with good oncological and functional Results.

**F67 Reconstruction of the Upper Limb by Vascularized Fibula, After Resection for Tumour, in Children and Adolescent**

E.J. Mascard[1], M. Germain[2], L. Wattincourt, J.M. Guinebretière, L. Brugières, J. Dubousset[2]
[1]*Hospital Saint-Vincent de Paul, Paris, France,* [2]*Institut Gustave Roussy, Villejuif, France*

The aim of our study was to report the Results and advantages of bone reconstruction by vascularized fibula in oncologic surgery of the upper-limb. From 1994 to 2000, ten patients underwent reconstruction of the upper-limb by vascularized fibula. There were 7 boys and 3 girls, aged 7 to 17. Histology was osteosarcoma in 7 cases, one Ewing, one low-grade osteosarcoma and one chondroma. Six patients received chemotherapy at time of the reconstruction. The vascularized fibula transfer was indicated in salvage of a failed humerus prosthesis in 2 patients, and as a primary procedure in 8, in whom 4 patients had resection-reconstruction of the radius and 4 from the humeral diaphysis. The mean graft length was 14.2 cm. Osteosynthesis was achieved by plate in most cases. In two cases, the peroneal artery was used to bypass the radial artery which was resected with the tumour. Results were retrospectively assessed after a 3.9 year mean follow-up (1 to 7). Nine out ten patients were free of disease. Complete bone healing of the vascularized fibula occurred after 3 months in average (1.5 to 5). Three fractures occurred after trauma, in diaphyseal humeral reconstruction and healed after revision. One radius reconstruction was revised for cancellous bone grafting. Progressive thickening of the graft allowed hardware removal in humerus reconstruction, only in younger children. In most of the radius reconstruction, the radiographic result was excellent. The mean final overall MSTS score was 85% (70 to 100). Vascularized transfer of the fibula offers an effective skeletal reconstruction of the upper limb after tumour-resection. Most of mechanical complication occurred in adolescents, and should have been avoided by limiting their sports activities. In humeral diaphyseal and radial intercalary reconstruction, functional Results were close to normal, and should be considered as definitive.

**F68 Proximal Humerus Reconstruction by Prosthesis Suspended to the Acromion after Tumour Resection, in Children and Adolescent**

E.J. Mascard[1], G. Lorton, P. Wicart, J.M. Guinebretière, L. Brugières, J. Dubousset[2]
[1]*Orthopedic Surgery Dept., Hospital Saint-Vincent de Paul, Paris, France,* [2]*Institut Gustave Roussy, Villejuif, France*

Post-operative dislocation is one of the commonest pitfalls in prosthetic reconstruction. The goal of our study was to report our experience in shoulder reconstruction by humeral prosthesis suspended to the acromion. From 1983 to 1999, 23 patients with bone tumours of the proximal humerus were treated, by resection and reconstruction with a prosthesis suspended to the acromion. There were 14 girls and 9 boys, aged 7 to 17 (mean 14.4). In one case the prosthesis was implanted in revision of a failed allograft and in the 22 remaining cases in primary reconstruction. Histology was an osteosarcoma in 16 cases, Ewing tumour in 3, chondrosarcoma in one case and bone metastasis in 3. An extra-articular resection (type VB) was performed in 8 cases, in the remaining cases an intra-articular (eleven 1B and four 1A) resection was performed. In all patients the prosthesis was suspended to the acromion with two non-absorbable braids. Post-operatively the shoulder was immobilized for three to four weeks. Results were retrospectively assessed with a 6.2 years mean follow-up (1 to 17). Three patients were lost-to follow-up, and 6 deceased from disease. There was no local recurrence. One patient developed a late infection that was treated by ablation of the prosthesis. Two prostheses were revised for loosening of the stem, with reconstruction by vascularized fibula. One prosthesis was revised for wear of the polyethylene humeral head. Two patients were revised for rupture of the non-absorbable braid. There was no dislocation. The mean MSTS functional score was 71.6%. In spite of limited active abduction and strength most of the patients felt satisfied. Reconstruction of the proximal humerus by prosthesis suspended to the acromion, offers acceptable functional Results, with few complications. For us, this simple and reliable technique remains indicated, when the axillary nerve and the abductor mechanism are involved.

**F69 Comparison of Unconstrained Versus Constrained Proximal Humeral Endoprosthesis for Primary Osteosarcoma**

J Borg[1], S Mahroof[2], J Pringle[3], P Unwin[2], TWR Briggs[2], SRC Cannon[2]
[1]*Royal National Orthopaedic Hospital, London, United Kingdom,* [2]*RNOH, Stanmore, London, United Kingdom,* [3]*Royal National Orthopaedic Hospital NHS, London, United Kingdom*

Endoprosthetic replacement of the proximal humerus is now accepted as the best treatment for many primary destructive lesions of the proximal humerus. However, due to the complication of migration of the prosthesis, there has been a change in the design from an unconstrained to a constrained device. Since 1978, 85 cases of endoprosthetic limb salvage surgery have been undertaken in our institution for a number of pathologies. These include osteosarcoma (27), chondrosarcoma (24), metastatic tumours (10), giant cell tumours (5), Ewing's sarcoma (4), malignant fibrous histiocytoma (3) and others (12). We reviewed the outcome of the patients treated for primary osteosarcoma. These patients are generally known to have a poor prognosis. Outcome was assessed using the upper extremity version of the Toronto Extremity Salvage Score. The majority of tumours were diagnosed by needle biopsy. There were 27 cases with 20 males and 7 females. The age at surgery ranged from 7 years to 70 years with the mean age being 21 years. All patients had preoperative chemotherapy. In 85% of tumours complete resection with good margins was possible while the rest were reported as having a focal marginal resection. There were 6 cases of local recurrence, 1 case of dislocation and two cases of deep infection, 1 early and one late. 8 patients died and in all cases pulmonary metastasis were present. In all cases there was retention of good elbow and hand function.

**F70 Tumours of the Distal Radius Surgical Anatomy and Principles of Resection and Reconstruction**

J. Bickels[1], J.C. Wittig[2], Y. Kollender[1], R. Oren[2], K. Kellar-Graney[2], M.M. Malawer[2], I. Meller[1]
[1]*Nat. Unit of Orthopedic Oncology, Tel Aviv Sourasky Medical Center, Tel Aviv, Israel,* [2]*Dept. of Othopedic Surgery, Washington Cancer Institute, Washington, DC, USA*

*Introduction:* The distal-radius is a difficult area in which to perform a tumour-resection due to its proximity to the wrist and the intimate relationship to the neurovascular-bundles of the hand. The current study summarize the principles of tumour-resection at the distal-radius and emphasizes the surgical-anatomy, principles of resection and reconstruction, and functional outcome.
*Methods:* Between 1982 and 2000 the authors treated 22 patients who had a tumour at the distal-radius. Diagnoses included 5 primary-bone-sarcomas, 3 soft-tissue sarcomas, and 14 benign-aggressive tumours. Sarcomas around the distal-radius were treated with wide resection of the distal-radius. Reconstruction utilized a vascularized-fibular-graft. Five of these patients underwent arthrodesis of their fibulocarpal-joint and a functional joint was preserved in the remaining three. Benign-aggressive tumours were treated with intralesional-curettage, burr-drilling, and cryosurgery. The wrist joint remained intact and reconstruction was performed with subchondral bone graft, cement, and hardware. All patients were followed for a minimum of 2 years.
*Results:* Patients who underwent intralesional tumour resection had an intact range-of-motion of the wrist joint. One of the three patients with a functional fibulocarpal joint had an intact range-of-motion and the remaining two had a 30° loss of wrist extension. Wrist motion was absent in the five patients who underwent wrist arthrodesis. Overall, 18 patients had good-to-excellent and 4 had moderate functional outcomes. Local tumour recurrence occurred in two patients with benign-aggressive tumour. These recurrences were treated satisfactorily with a second cryoablative procedure.
*Conclusion:* The biological behavior of a tumour of the distal radius dictates the margins of resection: primary sarcomas are treated with wide resection and benign-aggressive tumours are treated with intralesional resection. Good local tumour control associated with preserved range-of-motion and good functional outcome can be anticipated in the majority of these patients.

**F71 Enchondroma of the Hand Management with Curettage and Cemented Intramedullary Hardware**

J. Bickels[1], J.C. Wittig[2], Y. Kollender[1], R. Oren[2], K. Kellar-Graney[2], M.M. Malawer[2], I. Meller[1]
[1]*Nat. Unit of Orthopedic Oncology, Tel Aviv Sourasky Medical Center, Tel Aviv, Israel,* [2]*Dept. of Othopedic Surgery, Washington Cancer Institute, Washington, DC, USA*

*Introduction:* Surgical removal by means of curettage is the mainstay of treatment of enchondromas of the hand. Methods of reconstruction after tumour removal usually entail no reconstruction or filling of the tumour cavity with a bone graft, which necessitate a prolonged period of protected activity until bone healing occurs. The authors have utilized hardware and bone cement for the purpose of reconstruction. This technique provides immediate mechanical stability and allows early mobilization.

*Methods:* Between 1986 and 1999, the authors treated 13 patients who were diagnosed as having a solitary enchondroma of the hand. Anatomic locations included: metacarpal bones – 6, proximal phalanx – 4, and middle phalanx – 3. Surgery included exposure of the tumour through the retained thinned or destroyed cortex and removal of all gross tumour with hand curettes; this was followed by high-speed burr drilling of the inner reactive bone shell. Reconstruction included placement of an intramedullary metal wire and polymethylmethacrylate (PMMA). Full motion of the operated hand was allowed immediately after surgery. Unrestricted activity was allowed after 2 weeks. All patients were followed for more than 2 years.
*Results:* Following surgery, there were no neurovascular or tendon injuries, superficial or deep infections, or delayed stress fractures. All patients returned to their presurgical functional capability within two weeks. At the most recent followup, none of the patients had local tumour recurrence, residual swelling, or deformity. Seven patients had a mild loss of flexion at the interphalangeal joints; none considered this as having caused a functional limitation.
*Conclusion:* Reconstruction with intramedullary hardware and PMMA of the tumour cavity, remaining after curettage of enchondroma of the hand provides immediate mechanical stability and allows early mobilization. This technique is simple, safe, and associated with good functional outcome.

**F72 Endoprostetic Replacements of Distal Humeral Tumours: A Follow up of 31 Years**

A. Kulkarni, R.J. Grimer, S.R. Carter, R.M. Tillman
*The Royal Orthopaedic Hospital, Birmingham, United Kingdom*

*Aims:* Tumours of the distal humerus are rare but a challenge to treat. Options for treatment are excision and flail elbow, arthrodesis with considerable shortening, allograft replacement or endoprosthetic replacement (EPR). A retrospective analysis of 10 cases of EPR distal humerus was done to assess their success in treating tumours.
*Methods:* A retrospective analysis of 10 distal humeral tumours operated between 1970 and 2001 was done by retrieving data from notes. No patient was lost to follow up. The Toronto Extremity Salvage Score (TESS) was used to assess function in patients still alive.
*Results:* There were 4 male and 6 female patients, with ages ranging from 15 to 76 years. The period of follow up ranged from 5 months to 31 years. 8 patients had primary tumours and 2 had secondary tumours. 4 out of 10 patients died of metastatic disease 12 to 71 months after operation. None of the 10 patients had local recurrence, infection, amputation or nerve palsy. There were 3 revisions at 48, 56 and 366 months for aseptic loosening. There were 3 rebushings of the plastic inserts at 62, 78 and 113 months. Two of the three rebushings were done after revision of the humeral component at 6 months and 30 months. The average TESS Score for these patients was 72.91 (29.2 to 93.33).
*Conclusion:* Custom-made EPR for distal humeral tumours are an effective way of replacing the diseased bone leading to a reasonable level of function and an acceptable failure rate.

**F73 Is Resection-arthrodesis of the Humero-scapular Joint Better than Prosthetic Replacement for Tumours of the Proximal Humerus?**

B. Gunterberg, K. Berlin, P. Bergh
*Sahlgren University Hospital, Gothenburg, Sweden*

*Objective:* To assess the functional Results after resection-arthrodesis of the humero-scapular joint.
*Methods:* After resection of the proximal humerus for tumour, the defect (8–15 cm) was reconstructed with an autologous fibular graft (6 avascular, 2 vascular) and an iliac graft. The shaft of the fibular graft was inserted into the medullary canal of the humerus and the articular cartilage of the fibula and scapula was removed. The fibular graft was fixed to the scapula with a steel wire or an AO-plate. The iliac graft was interlocked between the humeral shaft and the coracoid process. Eight consecutive patients with a follow-up of 3–19, 5 years (average 10 years) were reviewed and function according to the MSTS-system was recorded.
*Results:* The mean age at diagnosis was 21 years. There were 3 chondrosarcomas, 2 osteosarcomas, 2 giant cell tumours, 1 aneurysmal bone cyst. At follow-up all patients were continuously disease-free. Four patients fractured their grafts. Two healed without problems, 1 had a painfree, useful pseud-arthrosis and 1 a disabling osteonecrosis-pseudarthrosis (the only irradiated patient). This patient had and MSTS-score of 17/30 (57%). The other 7 patients had MSTS-scores ranging from 25/30 (83%) to 29/30 (97%), average 27/30 (90%).
*Conclusion:* Good long-term Results can be achieved with humero-scapular resection-arthrodesis provided that radiotherapy is not given. In many cases prosthetic replacement appears not to be as functional as resection-arthrodesis.

**F74 Use of the Inverted Delta Shoulder Prosthesis in Reconstruction of the Proximal Humerus after Tumour Resection: Presentation of two series of six patients.**

G.M.L. Sys[1], L. De Wilde[1], Y. Julien[2], P. Trouilloud[2], D. Uyttendaele[1]
*[1]UZ Gent, GENT, Belgium, [2]Centre Universitaire, Dijon, France*

We present two series of six patients with a tumour of the proximal humerus, treated in two different centres by an inverted shoulder prosthesis type Delta (Depuy) after a Malawer type Ia or Ib resection. In one centre the resected part of the humerus was reimplanted after extracorporeal irradiation. It was fixed intramedullary by cementation of the humeral prosthetic component to allow easy restoration of humeral height. The largest glenosphere size 42 was routinely used to reconstruct the glenoid; this improves the functional outcome (increase of external rotation). In the other centre no graft augmentation except in one patient was used. The prosthetic stem was cemented in the remaining part of the humeral diaphysis. Stability of the prosthesis was directly related to the height of resection. The rationale behind the use of an inverted shoulder prosthesis is the improvement of the functional outcome in rotator cuff deficient shoulders. Mechanically this is established by the improvement of the deltoid muscle function by lengthening its lever arm through lowering and medialisation of the centre of rotation. This Results in a postoperative active abduction of at least 60 degrees, usually patients can perform abduction of at least 90 degrees. When compared to other Results found in literature this is a very large functional improvement. These Results are represented in the clinical evaluation by the Constant score: mean adjusted 70.3% (range: 32.6–82%).

**F75 Osteoarticular Allografts for Proximal Humeral Bone Tumours**

M. Donati, G. Bianchi, M. DiLiddo, H. DeGroot, M. Mercuri
*Istituti Ortopedici Rizzoli, Bologna, Italy*

We retrospectively reviewed the outcomes of 32 osteoarticular allografts in 31 consecutive patients who had intra-articular resection of the proximal humerus for a bone tumour. The average duration of follow-up was 5.3 years (range 2.4 to 10.8 years). Eleven patients had benign lesions and twenty had malignant tumours. Twenty-three of 31 allografts (72%) were filled with cement. Twenty-six patients were alive with no evidence of disease. Of the 30 reconstructions with more than 24 months follow-up, seven (23%) were revised or removed. Four of these seven were successfully converted to an allograft-prosthetic composite. Using removal or revision of the allograft as the end point, Kaplan-Meier analysis showed that the probability of survival of the reconstruction was 78% at 5 years. The average MSTS functional evaluation score was 22.4 out of thirty, representing 75% of expected normal function. The average range of motion was 49 degrees of forward flexion and 59 degrees of abduction. There were eleven allograft fractures. Allografts that were filled with cement had a lower incidence of fracture. Subchondral fractures were reduced in severity but not in number. Three joints dislocated, but two became stable without operative treatment. Nineteen of twenty patients who had been working prior to the procedure returned to work. Osteoarticular allografts remain a good choice for the reconstruction of the shoulder following S34A and S345A humeral resections. Filling the allografts with cement appears to reduce the incidence and severity of fractures.

**F76 Reconstruction Following Total Scapular Resection Analysis of Humeral Suspension Versus Endoprosthetic Reconstruction**

J. Bickels[1], J.C. Wittig[2], Y. Kollender[1], R. Oren[1], K. Kellar-Graney[2], M.M. Malawer[2], I. Meller[1]
[1]*Tel Aviv Sourasky Medical Center, Tel Aviv, Israel*
*2Washington Cancer Institute, Washington, USA*

*Introduction:* Total resection of the scapula has a major impact on shoulder function because of the sacrifice of the glenoid along with the stabilizing muscles of the shoulder. This impact is even more profound when there is an oncological necessity to resect the scapula with the opposing proximal humerus. The authors describe their experience with total scapular resections and two types of reconstructions, suspension of the proximal humerus from the clavicle with no reconstruction of the scapula, and endoprosthetic reconstruction of the scapula.
*Methods:* Between 1979 and 1998, the authors treated 23 patients who were diagnosed with 14 bone and 9 soft–tissue tumours and who required total scapular resection. Reconstruction included suspension of the remaining proximal humerus from the clavicle in 16 patients and endoprosthetic reconstruction of the scapula in 7 patients. Endoprosthetic reconstruction of the scapula was feasible only when the periscapular musculature was sufficiently preserved for endoprosthetic attachment and coverage. All patients were followed for a minimum of 2 years; the follow-up protocol included physical examination, radiological evaluation and functional evaluation according to the American Musculoskeletal Tumour Society system.
*Results:* Elbow range-of-motion and hand dexterity were similar in both groups. However, compared with patients who had undergone humeral suspension, those who had scapular endoprosthesis had better abduction of the shoulder joint and better cosmetic appearance of the posterior aspect of the shoulder girdle. The latter patients were far more likely to have a good-to-excellent functional outcome (86%) than the former (13%).
*Conclusion:* The number of patients in the current series does not allow for a valid statistical analysis; however, scapular endoprosthesis reconstruction appears to be associated with better functional and cosmetic outcomes than humeral suspension from the clavicle and is recommend by the authors when feasible.

**F77 Allograft/prosthetic Composite Reconstruction for Malignant Bone Tumours of the Proximal Humerus**

G.U. Exner[1], A.R. Von Hochstetter[2], Th.I. Malinin[3]
[1]*Univ. of Zurich, Zurich, Switzerland,* [2]*Pathologie Institut Enge, Zürich, Switzerland,* [3]*University of Miami, Miami, USA*

*Introduction:* Following curative resections of malignant tumours of the proximal humerus different options are available for the reconstruction (e.g. spacer/endoprosthesis, allograft, allograft/prosthetic compounds, autologous bone transfer, combinations) with different functional Results and long-term stability. We wish to report the Results after S3+S4 resections of the MST classification4.
*Methods:* 8 patients were treated by transarticular wide resection of the proximal humerus (1/3 to 2/3 of its total length) for osteosarcoma (3), Chondrosarcoma (2), irradiation sarcoma (1), mamma carcinoma metastasis (1), giant cell tumour (1). The reconstruction was performed with fresh frozen allografts cemented into custom revision prosthesis fixed cementless to the host bone. Tendons and ligaments of the rotator cuff and other preserved muscles were resutured to the corresponding structures of the allograft. Patients age ranged from 10 to 70 years. Follow-up is 4 to 7 years.
*Results:* No local recurrence occured. The patient with breast cancer died of metastases, the patient with irradiation sarcoma died of heart failure probably related to earlier irradiation, the 70 year old patient with an osteosarcoma lives with metastases at 6 years f/u, the others are disease free. All junctions healed. One shoulder has a habitual anterior instability. All patients have full internal rotation and can reach to the back and the forehead. Forward flexion of the shoulder ranges from 30 degrees to 150 degrees, average 80 degrees. External rotation is limited to 10 degrees to 30 degrees. The reconstruction is emotionally well accepted by all patients and they follow their pre-illness activities.
*Conclusion:* The reconstruction of proximal humerus resection with an allograft/prostetic compound has the potential of good functional Results and gives a high level of postoperative satisfaction.

**F78 Limb Salvage in Primary Malignant Humeral Tumours**

A.H.M. Taminiau, R.M. Bloem, J.L. Bloem, S. Dijkstra, P. Hogendoorn
*Leiden University Medical Center, Leiden, The Netherlands*

Limb salvage procedures in primary bone tumours of the humerus, if oncological adequate, offer attractive prospects regarding function of the shoulder elbow and hand. In humeral resections the axial nerve and deltoid muscle plays the key role in functional outcome.
*Methods:* From February 1979-February 1998 forty-five patients presenting with primary bone tumours of the humerus malignant 31 [chondrosarcoma (13), osteosarcoma (12), MFH (3), Ewing (2), and benign 14 [osteochondroma (6), GCT (5), ABC/osteoblastoma/fibr.dysplasia (all one)] were treated. Patient-gender [male (27), female (18)]. Mean-age 31,7 (10–79 y). Mean follow-up 12,3 years (3–23 y).
*Results:* 17 Patients had neo-adjuvant chemotherapy [osteosarcoma (12), MFH (3), Ewing(2)]. In 4 patients amputation was performed [fore-quarter (2), disarticulation (2)]. Forty-one patients had resections. Five patients without, thirty-six with reconstruction [allograft (19), allograft-prosthesis (11), vascularised fibula (4), prosthesis (2)]. Allograft procedures were osteoaticular (8), intercalary (6), inlay (5). Oncological-outcome: recurrence 2 (osteosarcoma, osteoblastoma), treated by re-resection (2). Survival: no evidence of disease: 35; tumour related death: 8 [osteosarcoma (4), MFH (2), chondrosarcoma (2)]; alive with disease: 2 (osteosarcoma). Resections without reconstruction (5) and inlay allografts (5) had all no complication and good function. Allograft-prosthesis composites (10) and prosthesis (2) showed moderate resorption of the tuberculum major (4), loosening (2), subluxation (2), infection (1), requiring revision surgery in 3. Osteoarticular and intercalary allografts (8/6) had fracture (2), pseudo-arthrosis (3) and were all surgically treated with bone-grafting or revision-allografting. Two of three osteoarticular allografts, showing joint detoriation, were resurfaced by prosthesis. Three infections [intercalary (2), allograft-prosthesis (1)] were revised by new allografts after treatment of the infection. No amputations were performed due to complications. Functional Results of intercalary allografts was good. Allograft-prosthesis composites and osteoarticular allografts had all but one stable shoulder joints, restricted motion in abduction and elevation and good hand/elbow function.

*Conclusion:* Limb salvage in humeral bone tumours is an acceptable procedure. Despite complications of allografts all upper limbs could be saved. The functional outcome if the joint was involved shows restricted but stable shoulder functions.

